# The American Dental Convention

**Published:** 1866-06

**Authors:** 


### THE AMERICAN DENTAL CONVENTION.

Will meet in the city of New York on the first Tuesday of August. We anticipate a large gathering of the profession at that time, if no adverse circumstances arise.

In the free and unlimited discussion and interchange of opinion granted in this body much good results. It is eminently adapted for the cultivation of professional acquaintance and sociability. We say to all, attend the American Dental Convention. It will as it always has benefit all who attend.

